# Two new non-spiny *Solanum* species from the Bolivian Andes (Morelloid Clade)

**DOI:** 10.3897/phytokeys.47.4423

**Published:** 2015-03-20

**Authors:** Tiina Särkinen, Sandra Knapp, Michael Nee

**Affiliations:** 1 Royal Botanic Garden Edinburgh, 20A Inverleith Row, EH3 5LR Edinburgh, United Kingdom; 2 Department of Life Sciences, Natural History Museum, Cromwell Rd, SW7 5BD London, United Kingdom; 3 Missouri Botanical Garden, P.O. Box 299, St. Louis, MO 63166-0299, U.S.A.

**Keywords:** Bolivia, endemism, Morelloid Clade, tropical Andes, Solanaceae, Solanum section Solanum, Andes tropicales, Bolivia, Clado Morelloid, endemismo, Solanaceae, Solanum sección Solanum

## Abstract

Two new Bolivian species are described from the Morelloid clade of *Solanum* (section *Solanum* in the traditional sense). *Solanum alliariifolium* M.Nee & Särkinen, **sp. nov.** is found in montane forests between 1,900 and 3,200 m and is morphologically most similar to *Solanum leptocaulon* Van Heurck & Müll.Arg., also from montane forests in southern Peru and Bolivia. *Solanum rhizomatum* Särkinen & M.Nee, **sp. nov.** is found in seasonally dry forests and matorral vegetation in lower elevations between 1,300 and 2,900 m and is most similar to *Solanum pygmaeum* Cav., a species native to sub-tropical Argentina but introduced in subtropical and temperate areas worldwide.

## Abstract (es)

Se describen dos nuevas especies bolivianas del clado Morelloid de *Solanum* (sección *Solanum* en sentido tradicional). *Solanum alliariifolium* M.Nee & Särkinen, **sp. nov.** se encuentra en bosques montanos entre 1,900 y 3,200 metros y es morfológicamente muy similar a *Solanum leptocaulon* Van Heurck & Müll.Arg., también presente en bosques montanos en el sur de Perú y Bolivia. *Solanum rhizomatum* Särkinen y M.Nee, **sp. nov.** se encuentra en bosques tropicales estacionalmente secos y en vegetación de matorral a elevaciones más bajas entre 1,300 y 2,900 m, y es más similar a *Solanum pygmaeum* Cav., una especie nativa para Argentina subtropical pero introducida en las zonas subtropicales y templadas del mundo.

## Introduction

*Solanum* is one of the most species-rich vascular plant genera in the tropical Andes (Jørgensen et al. 2011), where many new species continue to be described (e.g., Anderson et al. 2006; Stern and Bohs 2010; Knapp 2010a,b; Farrugia and Bohs 2010; Tepe et al. 2012; Särkinen et al. 2013a; Särkinen et al. 2015). Here we describe two new *Solanum* species from Bolivia that belong to the Morelloid clade, one of major clades of non-spiny solanums (Weese and Bohs 2007; Särkinen et al. 2013b).

The Morelloid clade is a group of ca. 75 species, most of which are endemic to the tropical Andes (Bohs 2005; Särkinen et al. in review). The clade includes five major groups (traditionally recognised as sections *Solanum*, *Campanulisolanum* Bitter, *Parasolanum* A.Child, *Chamasarachidium* Bitter, and *Episarcophyllum* Bitter), which are in the process of re-circumscription based on molecular results (Särkinen et al. in review). Section *Solanum* is the largest of these with ca. 52 species and ca. 580 published names and is the only group to occur outside of the Americas. The Morelloid clade is distinguished by its herbaceous or sub-shrubby habit, usually internodal inflorescences, small flowers and fruits, and the usual possession of stone cells in the fruits (Bitter 1911). These are sclerified structures that are usually white and spherical rather than flattened and brown or yellowish brown like the seeds. Although some studies have examined the taxonomy of the Old World and North American species of this group (Edmonds 1977, 1978; Schilling 1981), monographic treatment is needed to aid species identification and to clarify synonymy in South America, where most of the species diversity is found (Edmonds 1972; Barboza et al. 2013).

Recent taxonomic work focusing on producing a monographic treatment of the Morelloid clade has resulted in the description of various new species from Peru, Bolivia and Ecuador (Särkinen et al. 2013a; Särkinen et al. 2015). Two additional new species are described here from Bolivia. Descriptions are based on field work and examination of herbarium specimens from 20 herbaria (BH, BM, COL, CORD, CPUN, DUKE, E, F, GH, GOET, HUSA, HUT, K, LPB, MO, MOL, NY, S, UDBC, US, USM, USZ). Extent of Occurrence (EOO) and Area of Occupancy (AOO) were calculated using GeoCat (www.geocat.kew.org) with a 2 km^2^ cell size for AOO calculation. Conservation status of each species was assessed using the IUCN (2014) criteria based on the GeoCat analyses (Bachman et al. 2011) combined with field knowledge. All specimens are cited in the text, and full data is provided in the Suppl. material 1 and on Solanaceae Source (www.solanaceaesource.org).

## Taxonomic treatment

### Solanum alliariifolium

Taxon classificationPlantaeSolanalesSolanaceae

M.Nee & Särkinen sp. nov.

urn:lsid:ipni.org:names:77145835-1

Figs 1 , 2

#### Diagnosis.

Similar to *Solanum leptocaulon* Van Heurck & Müll.Arg., but differing in its slender creeping habit with stems rooting along nodes, broadly ovate to orbicular leaves with mostly undulate to shallowly lobed margins, and a stellate corollas lobed to the middle with lobes reflexed at anthesis.

#### Type.

**Bolivia. Santa Cruz.** Prov. Vallegrande: 6.5 km by air SW of Guadalupe on rd to Pucará, at turnoff to Santa Ana, 18°36'S, 64°07'W, 2675 m, 15 Dec 1990 (fl,fr), *M. Nee 40315* (holotype: LPB; isotypes: MO [MO-2537105], NY [NY00852828], USZ).

#### Description.

Slender herb to 20–30 cm high, with multiple long, creeping stems arising from a central taproot. Stems rooting at nodes, 1–2 mm in diameter, up to 50 cm long, glabrous or sparsely pubescent with spreading translucent 4–6-celled simple uniseriate trichomes ca. 0.2 mm long. Sympodial units difoliate, not geminate. Leaves simple, 1.5–3.6 cm long, 0.9–2.3 cm wide, broadly ovate to orbicular; adaxial surface glabrous; abaxial surface glabrous or sparely pubescent with appressed 1–3-celled simple uniseriate trichomes along veins and leaf margins; primary veins 3–4 pairs; base rounded to attenuate, occasionally decurrent; margins entire, undulate, or shallowly lobed; apex acute; petiole 0.7–1.5 cm long, sparsely pubescent with simple 1–3-celled uniseriate trichomes like those of the stems, especially on young leaves. Inflorescences 1.5–3.0 cm long, simple, lateral, leaf-opposing or internodal, with 2–6 flowers, sparsely pubescent with simple uniseriate 4–6-celled spreading trichomes; peduncle 1.0–3.0 cm long, 0.4–0.5 mm in diameter at the apex and 0.6 mm in diameter at the base; pedicels 0.6–0.9 cm long, ca. 0.4 mm in diameter at the base and ca. 0.5 mm in diameter at the apex, straight and spreading at anthesis, articulated at the base; pedicel scars spaced 0.2–1.5 mm apart. Buds globose, white or purple-tinged. Flowers 5-merous, all perfect, nodding; calyx tube ca. 1.4–1.5 mm long, the lobes 1.6–2.0 mm long, rectangular-deltate in outline with rounded to acute apices, somewhat spreading at anthesis, sparsely pubescent with simple 1–4-celled uniseriate trichomes; corolla 1.4–1.6 cm in diameter, white to pale or deep violet-blue, with a dark purple ring and yellow-green central star at the base, stellate, lobed to the middle, the lobes ca. 4.0–5.0 mm long, 2.0-2.5 mm wide, reflexed at anthesis, densely pubescent abaxially with 1–2-celled simple uniseriate trichomes, these usually shorter than the trichomes of stems and leaves, glabrous adaxially; filament tube 1.3–1.5 mm long; free portion of the filaments ca. 1.1–1.6 mm long, pubescent with 4–7-celled uniseriate trichomes at the base adaxially; anthers 3.5–4.0 mm long, 0.8–1.0 mm wide, ellipsoid to rectangular in outline, yellow, poricidal at the tips, the pores lengthening to slits with age; ovary globose, glabrous; style 5–6 mm long, exerted 1.0–1.7 mm beyond the anther cone, densely pubescent with 2–3-celled simple uniseriate trichomes in the basal 2/3; stigma clavate, minutely papillate. Fruit a globose berry, 4–5 mm in diameter, green when developing, the colour when mature unknown, with a few stone cell aggregates in each berry; fruiting pedicels 1.1–3.2 cm long, ca. 0.4 mm in diameter at the base, ca. 0.6 mm in diameter at the apex, spreading, becoming somewhat woody; fruiting calyx lobes 2.8–3.2 mm long, spreading. Seeds 15–20 per berry, ca. 1.5–1.7 mm long, ca. 1.2–1.3 mm wide, flattened, reniform, pale-brown, the sub-lateral hilum positioned close to the middle, the testal cells pentagonal in outline.

**Figure 1.**
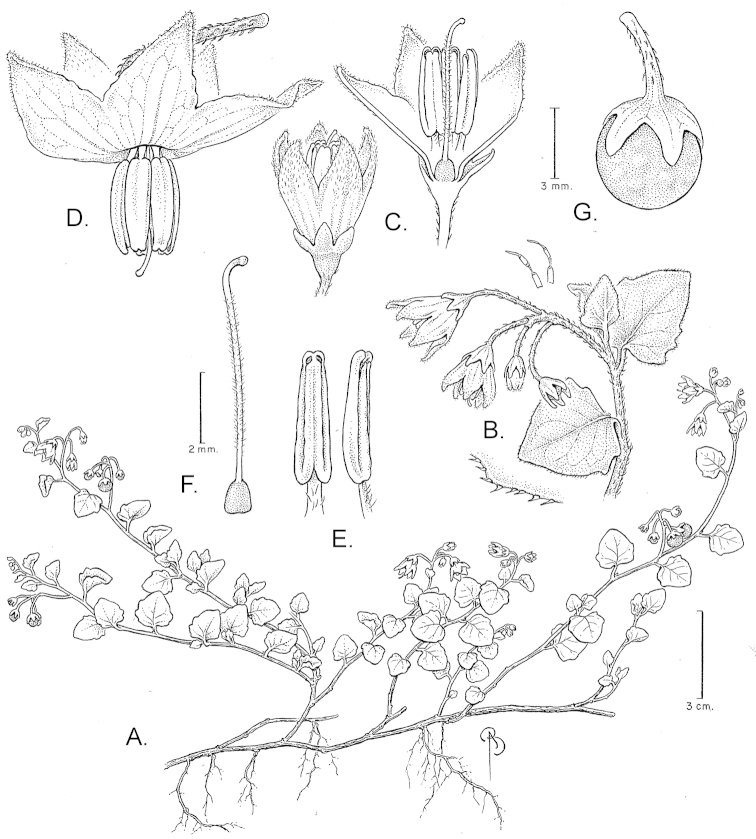
Illustration of *Solanum alliariifolium*. **A** Habit **B** Inflorescence with details of indumentum of simple, multi-cellular hairs along the stem, and short ciliate hairs along leaf margins **C** Flower just before anthesis, with and without corolla lobes removed **D** Flower at anthesis **E** Stamens **F** Gynoecium **G** Fruit (**A–C, E–G** *Nee 40315*, **D** *Vargas 787*). Illustration by Bobbi Angell.

#### Distribution.

Endemic to montane forests of the Eastern Bolivian Andes in the Departments of Chuquisaca, Cochabamba, and Santa Cruz, in open areas close to water sources, near rivers and moist depressions, and marshy meadows on sandy or rocky substrates, associated with *Podocarpus parlatorei* Pilg., *Alnus acuminata* Kunth, *Hesperomeles ferruginea* (Pers.) Benth., *Alchemilla pinnata* Ruiz & Pav., *Azorella biloba* (Schltdl.) Wedd., *Weinmannia fagaroides* Kunth, *Baccharis genistelloides* (Lam.) Pers., *Clethra scabra* Pers., *Myrsine coriacea* (Sw.) Roem. & Schult., *Symplocos nana* Brand, *Eleocharis* spp., *Chusquea* spp., *Morella pubescens* (Willd.) Wilbur, ferns, grasses and Apiaceae herbs; between 1,900 and 3,200 m elevation.

**Figure 2.**
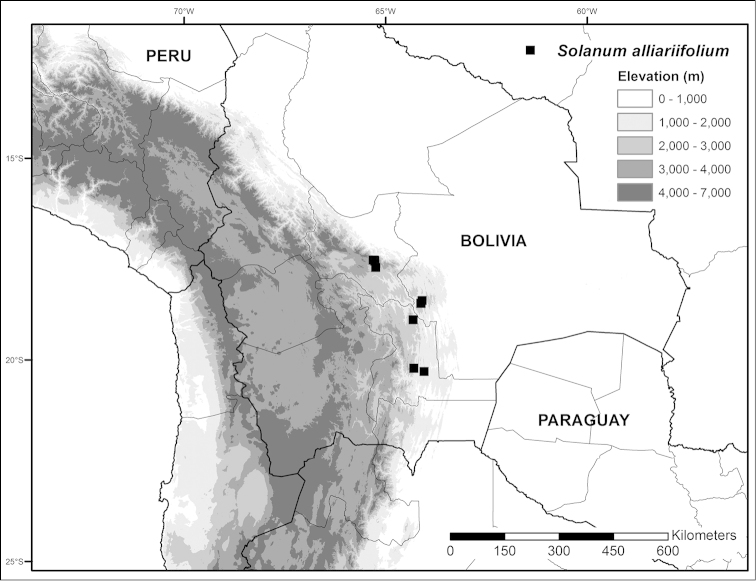
Distribution map of *Solanum alliariifolium*.

#### Ecology.

Flowering and fruiting during the wet season, generally from October-March, with a single record known from August.

#### Etymology.

The epithet refers to the leaf shape, which struck the collector of the type (MN) as like that of *Alliaria petiolata* (M.Bieb.) Cavara & Grande (Brassicaceae), a European species invasively adventive in the eastern USA and other temperate areas of the world.

#### Conservation status.

We assign a preliminary IUCN threat status of Vulnerable (VU, B1) to *Solanum alliariifolium* based on the small extent of occurrence (EOO=16,136 km^2^). The area of occupancy is even smaller (AOO=40 km^2^) and would merit status as endangered (EN), but knowing that collection densities in the tropical Andes remain extremely low and that the collections are mainly along the sparse road network, we prefer basing our assessment on the extent rather than area of occurrence. No occurrences are known within the protected area network in Bolivia thus far, but collection data indicates that the species endures grazing pressures relatively well.

#### Specimens examined.

**Bolivia. Chuquisaca: Prov. Belisario Boeto:** ca. 5 km S of Nuevo Mundo at summit of rd to Villa Serrano, 2,300 m, 18 Oct 1997, *J.R.I. Wood 12710* (K). **Prov. Hernando Siles:** Primera Sección Monteagudo, Cantón Fernández, Comunidad Vallecito, 20°12'43"S, 64°18'03"W, 2,314 m, 11 Aug 2007, *M. Jiménez 603* (NY); Laguna Milagros, 20°17'16"S, 64°02'56"W, 1,993 m, 26 Dec 2005, *R. Lozano & M. Serrano 1787* (MO, NY); Primera Sección Monteagudo, Cantón Fernández, Comunidad Vallecito, 20°12'18"S, 64°17'58"W, 2,456 m, 9 Nov 2007, *J. Villalobos 927* (MO). **Cochabamba: Prov. Carrasco:** Siberia, 17°49'87"S 64°44'07"W, 2,940 m, 20 Feb 2005, *S. Altamirano & M. Alcázar 3075* (MO); Sehuenka–Totora, 2,800 m, Nov 1959, *M. Cárdenas 5716* (US); Jatum Pino, 3200 m, Jan 1961, *M. Cárdenas 5942* (K); near Lagunillas (Totora), 2,700 m, Jan 1951, *M. Cárdenas 4663* (US); narrow canyon of Rio Monte Puncu, 5 km NE of Monte Puncu, 10 km by air NW of Epizana, 17°33'S, 65°16'W, 2,700– 2,750 m, 10 Mar 1988, *M. Nee 36631* (NY); de Episana a Sehuancas via Montepunca, 2,500 m, 25 Mar 1978, *C.M. Ochoa 12022* (US); 5 km above Sehuencas towards Monte Puncu, 2,500 m, 4 Feb 1995, *J.R.I. Wood 9317* (K). **Santa Cruz: Prov Caballero:** 5.5 km (by rd) E of town of El Churro at pond along Pojo–Comarapa highway, 17°50'15"S 64°44'00"W, 2,940 m, 22 Jan 2006, *M. Nee & Jun Wen 53903* (MO, USZ). **Prov. Vallegrande:** Huasacañada, 5 km al S de la ciudad de Vallegrande, 18°31'30"S 64°05'42"W, 2,050 m, 3 Nov 1990, *I. Vargas C. 787* (MO, NY, USZ); carretera entre Vallegrande y Pucará, 18°35'49"S 64°07'34"W, 2,673 m, 5 Mar 2005, *J.R.I. Wood 21774* (K, USZ).

#### Discussion.

*Solanum alliariifolium* is distinct within the Morelloids in being a slender creeping herb rooting at the nodes, with broadly ovate to orbicular leaves with undulate to shallowly lobed margins. It is morphologically most similar to *Solanum leptocaulon* Van Heurck & Müll.Arg., which occurs in similar montane habitats in Bolivia and in southern Peru, but the latter species is a small scrambling shrublet with ovate-lanceolate leaves with entire margins. *Solanum leptocaulon* further differs from *Solanum alliariifolium* in having a campanulate corolla lobed only 1/3 of the way to the base, rather than a stellate corolla lobed to 2/3 to the base with the lobes clearly reflexed at anthesis.

### Solanum rhizomatum

Taxon classificationPlantaeSolanalesSolanaceae

Särkinen & M.Nee sp. nov.

urn:lsid:ipni.org:names:77145836-1

Figs 3 , 4

#### Diagnosis.

Like *Solanum pygmaeum* Cav., but differing in having mostly 1-branched inflorescences with 6–15 flowers, anthers < 3.5 mm long, strongly recurving fruiting pedicels, and berries < 1 cm in diameter with fewer than 30 seeds.

#### Type.

**Bolivia. Santa Cruz:** Prov. Vallegrande, 10 km (by air) NNW of Vallegrande, 18°23'S, 64°08'W, 1850 m, 1 Feb 1987, *M. Nee & G. Coimbra S. 33947* (holotype: LPB; isotypes: G, MO [MO-5894880], NY [NY00824501]).

#### Description.

Rhizomatous herb with erect stems up to 15–50 cm tall arising from an underground rhizome. Stems 1.5–4.0 mm in diameter at base, slightly flexuose, terete to ridged, often slightly winged, often purple-coloured, glabrous to sparsely pubescent with appressed 1–4-celled simple uniseriate trichomes ca. 0.5 mm long. Sympodial units difoliate, not geminate. Leaves simple, 2.3–8.0 cm long, 1.2–4.3 cm wide, ovate-lanceolate; adaxial surface glabrous or sparsely pubescent with 1–2-celled spreading hairs along lamina and veins; abaxial surface pubescent only along veins; primary veins 4–6 pairs; base attenuate to decurrent; margins lobed to entire, often purple-tinged, pubescent with short, 1-celled simple uniseriate trichomes, if present lobes present throughout or most commonly only in the basal 1/3 of the blade; apex acute to acuminate; petiole 0.5–1.2 cm long, sparsely pubescent with spreading, simple uniseriate trichomes like those of the stems and leaves. Inflorescences 1.5–3.1 cm long, lateral and internodal, simple to 1-branched, with 6–15 flowers, sparsely pubescent with simple 1–4-celled uniseriate appressed trichomes; peduncle 1.0–2.4 cm long, and if branched, each branch with a rachis 3–4 mm long; pedicels 4–6 mm long, ca. 0.3 mm in diameter at the base and ca. 0.4 mm in diameter at the apex, straight and spreading at anthesis, articulated at the base; pedicel scars spaced 1–2 mm apart. Buds ovoid, white or purple-tinged. Flowers 5-merous, all perfect; calyx tube ca. 2.0–2.5 mm long, the lobes 1.0–1.5 mm long, triangular with acute apices, sparsely pubescent with simple 1–3-celled appressed uniseriate trichomes; corolla 1.2–1.5 cm in diameter, white or flushed with blue, with a yellow-green basal star, stellate, lobed 1/2 to 2/3 of the way to the base, the lobes 4.0–5.0 mm long, 2.5–3.0 mm wide, reflexed at anthesis, later spreading, densely pubescent abaxially with 1–2-celled simple uniseriate trichomes, these usually shorter than the trichomes of stems and leaves, glabrous adaxially; filament tube 1.2–1.5 mm long; free portion of the filaments 1.0–1.2 mm long, pubescent along internal side with spreading hairs like those of the stems and leaves; anthers 3.2–3.5 mm long, 0.9–1.0 mm wide, ellipsoid or rectangular in outline, yellow; ovary globose, glabrous; style 6–7 mm long, exerted 2.5–3.0 mm beyond the anther cone, densely pubescent with 4-celled simple uniseriate trichomes in the basal 2/3; stigma globose, minutely papillate. Fruit a globose berry, 6–7 mm in diameter, pale green (mature ?), with a few stone cell aggregates; fruiting pedicels 1.2–1.4 mm long, ca. 0.6 mm in diameter at the base, ca. 0.8 mm in diameter at the apex, strongly recurving; fruiting calyx lobes 2.5–3.5 mm long, appressed to the berry with the tips slightly reflexed. Seeds 15–25 per berry, 1.7–1.8 mm long, 1.4–1.5 mm wide, concave-reniform, pale brown, the hilum positioned towards the narrower end of the seed, the testal cells pentagonal in outline.

**Figure 3.**
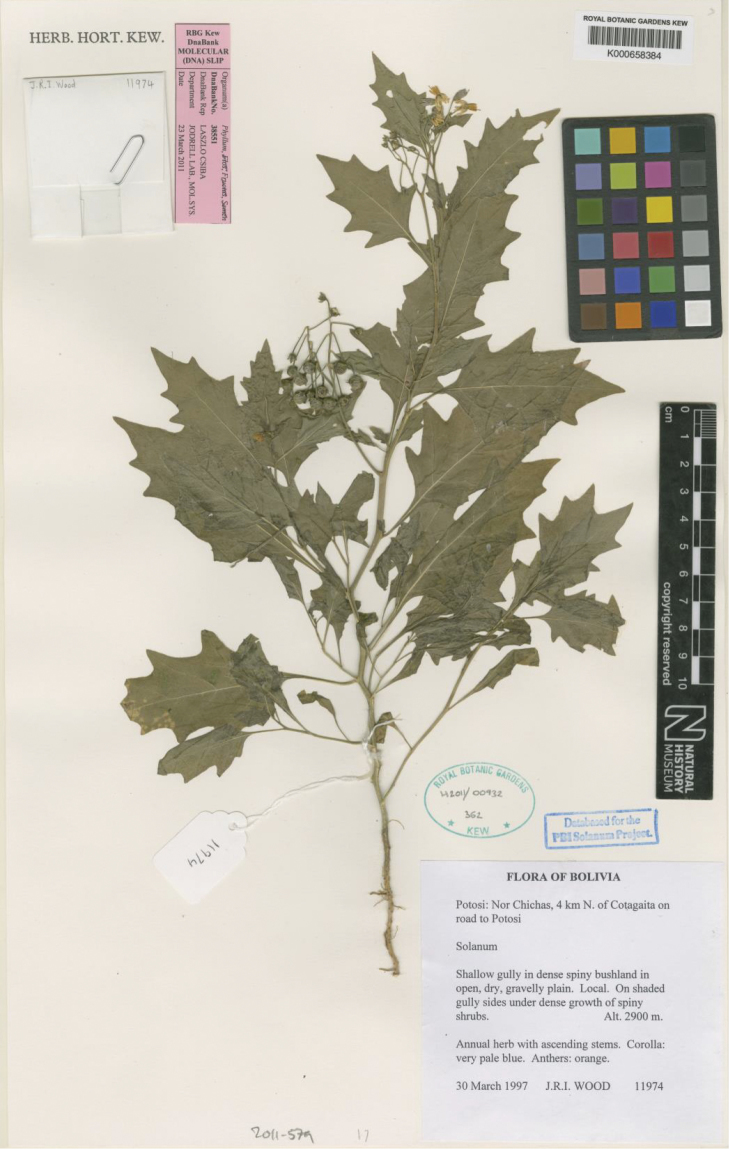
Paratype of *Solanum rhizomatum* (*Wood 11974*, K).

**Figure 4.**
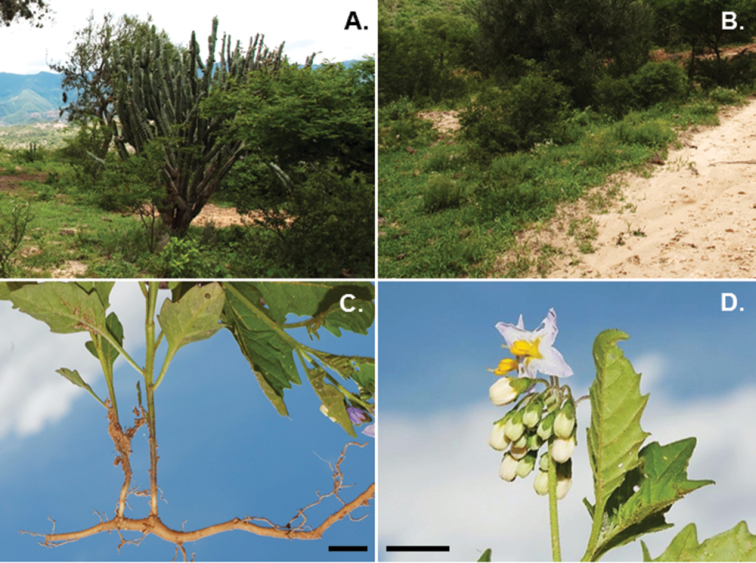
*Solanum rhizomatum*. **A** Habitat in seasonally dry forests, eastern Bolivian Andes, Vallegrande, Dept. Santa Cruz **B** Habit amongst low herbs in partial shade **C** Rhizome **D** Inflorescence (**A–D** *Nee & Mendoza 57594*). Photos by M. Nee, scale bars = 1 cm.

#### Distribution.

Endemic to the arid interior valleys of the Bolivian Andes in the Departments of Cochabamba, Potosí, Santa Cruz, and probably Chuquisaca, growing in seasonally dry tropical forests and dry matorral vegetation, along slopes and on rocky and sandy soils, often found growing in moist depressions under the shade of larger trees and thickets, associated with *Prosopis kuntzei* Harms ex Kuntze, *Jodina rhombifolia* (Hook. & Arn.) Reissek, legumes, grasses, columnar cacti, and Asteraceae herbs; between 1,300 and 2,900 m elevation.

**Figure 5.**
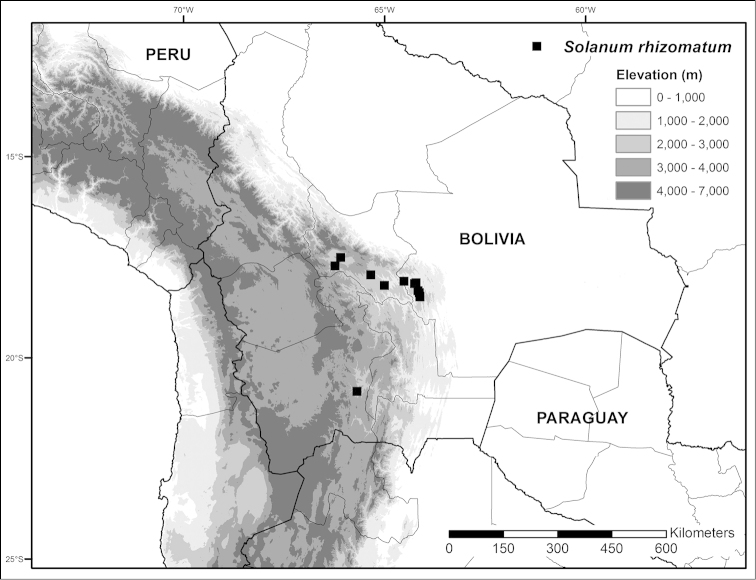
Distribution map of *Solanum rhizomatum*.

#### Ecology.

Flowering and fruiting during the wet season from Jan. to March.

#### Etymology.

*Solanum rhizomatum* is named for its rhizomatous underground stem.

#### Conservation status.

We assign a preliminary IUCN threat status of Least Concern (LC) to *Solanum rhizomatum* based on the known extent of the species occurrence (EOO=43,101 km^2^). The extremely small observed area of occupancy (AOO=48 km^2^) could merit endangered status (EN), but knowing that collection densities in the tropical Andes remain extremely low and considering that current collections are from >10 different localities, we prefer basing our threat status assessment on the extent rather than area of occurrence. It is not known whether *Solanum rhizomatum* is similar in its biology and vegetative spread to *Solanum pygmaeum*, and further studies may clarify this aspect of potential conservation assessments in the future. No populations are known thus far from the protected area network in Bolivia. The growth form that allows effective vegetative spreading would indicate that the species can withstand grazing pressures moderately well.

#### Specimens examined.

**Bolivia. Cochabamba: Prov. Campero:** Mizque, 2,020 m, 20 Feb 1967, *R.F. Steinbach 721* (US); ca. 5 km de Villa Granada hacia Peña Colorada, 18°12'10"S, 65°00'09"W, 2,201 m, 25 Feb 2004, *J.R.I. Wood 20266* (K, LPB). **Prov. Cercado:** en la salida de Cochabamba hacia el Valle Alto pasando La Tranca y ca. 2 km antes de la Angostura, 17°30'24"S 66°05'35"W, 2,676 m, 10 Feb 2005, *J.R.I. Wood 21590* (K). **Potosí: Prov. Nor Chichas:** 4 km N of Cotagaita on road to Potosí, 20°50'S, 65°41'W, 2,900 m, 30 Mar 1997, *J.R.I. Wood 11974* (K, LPB). **Santa Cruz: Prov. Caballero:** carretera de Pulquina a Saipina a 0.5 km al oeste de la cumbre, 18°05'58"S, 64°30'56"W, 1,788 m, 20 Feb 2003, *J.R.I. Wood 19134* (BOLV, K). **Prov. Florida:** 3 km S of Mataral, 18°08'S, 64°13'W, 1,425 m, 6 Feb 1988, *M. Nee 36252* (MO, NY, USZ); 4 km by road W of Mataral, 18°09'21"S, 64°15'17"W, 1,300 m, 22 Feb 1984, *G. Schmitt 27A* (MO). **Prov. Vallegrande:** Pueblo de Vallegrande, Cerro los Tres Pilares, 100–150 m antes de llegar a la cima sobre el sendero hacia el pueblo, 18°29'21"S, 64°07'13"W, 2,236 m, 19 Jan 2003, *M. Mendoza 449* (K); Choroquetal, ca. 5 km de Vallegrande sobre la carretera a Mataral, entrando ca. 500 m, sobre la senda hacia Chacateal, 18°28'07"S, 64°07'25"W, 1,932 m, 17 Mar 2003, *M. Mendoza 529* (K); 4 km SW El Trigal, 18°20'S, 64°10'W, 1,600 m, 8 Mar 1988, *M. Nee 36536* (MO, NY, USZ); Las Cañas, 2500 m, 28 Feb 1984, *C.M. Ochoa 15548* (US); 6.5 km (by air) NE of airport in Vallegrande, along bad dirt road down into the Río San Blas valley, 18°26'33"S 64°03'12"W 1,795 m, 3 Jan 2011, *M. Nee & M. Mendoza 57594* (USZ).

#### Discussion.

*Solanum rhizomatum* is most closely related to *Solanum pygmaeum* from central and coastal Argentina (see Barboza et al. 2013), another rhizomatous species of Solanum section Solanum. *Solanum rhizomatum* differs from *Solanum pygmaeum* in having mostly 1-branched inflorescences with 6–15 flowers, anthers 3.2–3.5 mm long, strongly recurving fruiting pedicels, and berries with 15–25 seeds, while *Solanum pygmaeum* always has simple (unbranched) inflorescences with 2–6 flowers, anthers usually >3.5 mm long, fruiting pedicels that are broadly spreading, and berries with >50 seeds. Although these sets of characters overlap to some extent, *Solanum pygmaeum* individuals are generally smaller than those of *Solanum rhizomatum* (10–20 cm tall), with smaller leaves 1–5 cm long and 0.5–2.2 cm wide, while *Solanum rhizomatum* grows 15–50 cm tall, with larger leaves 2.3–8.0 cm long and 1.2–4.3 cm wide. *Solanum pygmaeum* forms dense colonies in secondary habitats such as railroad sidings, and has been introduced and naturalised in Europe and North America, presumably in boats carrying wool from eastern coastal Argentina (Barboza et al. 2013).

As in many species of *Solanum*, variation in corolla colour occurs in *Solanum rhizomatum*, where corollas vary from white to pale lilac even within single individuals. Label information from *Nee & Mendoza 57594* notes changes in the corolla colour during development, where the corolla is white in bud, violet in anthesis, and darker after wilting.

## Supplementary Material

XML Treatment for Solanum alliariifolium

XML Treatment for Solanum rhizomatum

## Supplementary materials

Supplementary material 1 Occurrence records of Solanum alliariifolium and Solanum rhizomatum Data type: occurence Explanation note: Occurrence records of the two new *Solanum* species. This dataset is made available under the Open Database License (http://opendatacommons.org/licenses/odbl/1.0/). The Open Database License (ODbL) is a license agreement intended to allow users to freely share, modify, and use this Dataset while maintaining this same freedom for others, provided that the original source and author(s) are credited.Tiina Särkinen, Sandra Knapp, Michael Nee
